# The Burden of Trachoma in South Sudan: Assessing the Health Losses from a Condition of Graded Severity

**DOI:** 10.1371/journal.pntd.0001538

**Published:** 2012-03-06

**Authors:** Hebe Gouda, John Powles, Jan Barendregt, Paul Emerson, Jeremiah Ngondi

**Affiliations:** 1 Institute of Public Health, University of Cambridge, Cambridge, United Kingdom; 2 School of Population Health, University of Queensland, Brisbane, Australia; 3 Trachoma Control Program, The Carter Center, Atlanta, Georgia, United States of America; University of California San Diego School of Medicine, United States of America

## Abstract

**Introduction:**

Trachoma is a disease that can lead to visual impairment and ultimately blindness. Previous estimates of health losses from trachoma using the Global Burden of Disease methodology have not, however, included the stage prior to visual impairment. We estimated the burden of all stages of trachoma in South Sudan and assessed the uncertainty associated with the severity and duration of stages of trachoma prior to full blindness.

**Methods:**

The prevalence of trachoma with normal vision, low vision and blindness in the Republic of South Sudan has been estimated previously. These estimates were used to model the incidence and duration of the different stages employing DISMOD II. Different assumptions about disability weights and duration were used to estimate the Years Lived with Disability (YLD).

**Results:**

We have estimated the total burden of trachoma in South Sudan to be between 136,562 and 163,695 YLD and trichiasis with normal vision contributes between 5% and 21% of the total depending on the disability weight applied. Women experience more of this burden than men. The sensitivity of the results to different assumptions about the disability weights is partly dependent upon the assumed duration of the different disease states.

**Interpretation:**

A better understanding of the natural history of trachoma is critical for a more accurate burden estimate.

## Author Summary

Trachoma is an infectious disease that is endemic to the Republic of South Sudan. In the absence of appropriate treatment recurrent re-infection in an individual will lead to progressively severe states of trachoma, eventually leading to the loss of visual acuity and finally blindness. Here we distinguish between three separate states of disease: trachoma with normal vision, trachoma with low vision and trachoma with blindness. The first of these states, trachoma with normal vision, is the least severe and the impact of this state on a population has not been well investigated. Trachoma, even before any loss of vision, comes with a great deal of pain and social consequences, and thus disability. In this study we employ data from South Sudan and estimate the burden caused by trachoma with normal vision for the first time. In doing so, we also reveal the extent of the gaps in our knowledge surrounding the natural history of trachoma and highlight areas of research that require urgent attention.

## Introduction

Trachoma is a neglected tropical disease that is endemic in the Republic of South Sudan and more than 50 other countries in the world [1]. Globally, trachoma is the leading infectious cause of blindness and the eighth most common blinding disease [2]. When susceptible individuals come into contact with the bacterium *Chlamydia trachomatis*, they may become infected. Recurrent re-infection by the bacterium can eventually lead to the repositioning of the eyelashes back towards the cornea, a condition known as trichiasis. Without treatment this state eventually begins to impair the individual's vision and ultimately leads to blindness.

Many neglected tropical diseases cause years of suffering. When the Global Burden of Disease methodology is used to assess the public health importance of chronic conditions such as trachoma, the resulting Disability Adjusted Life Years (DALYs) estimates are dominated by the estimated years lived with disability (YLD). The burden estimates will thus be sensitive to the disability weights applied to different health states. While this is an issue for all nonfatal conditions, it can be a particularly important issue for conditions that are highly prevalent with lower levels of severity [3]. This is because small absolute changes to the already small disability weights can make proportionately large differences to the overall estimate. Uncertainty about disability weights may therefore be most consequential where chronic, low severity conditions exist in large parts of the populations. This describes the case of trachoma in South Sudan.

Previous assessments of the global burden of trachoma have not included health losses from stages prior to visual impairment. Prior to the loss of visual acuity, however, trichiasis is associated with pain, photophobia and sensitivity to smoke and dust and therefore reduced capabilities in everyday life [4]. Frick et al [5], for example posited that trichiasis with normal vision may result in economic burden comparable to trachomatous low vision and one study has suggested that the inclusion of trichiasis with normal vision in the burden estimate could add up to 50% to the total burden of trachoma [6].

Between 2001 and 2005 Ngondi et al [7] collected data on visual acuity in the Mankien district providing the most reliable estimates of the prevalence of trichiasis with normal vision, low vision and blinding available for South Sudan. According to these observations and extrapolating to the whole of South Sudan, it is clear that many of those with trichiasis are not visually impaired and have thus not been included in the attempts to estimate the burden of trachoma.

The first aim of this study is to determine whether leaving out the least severe end of the disease spectrum is likely to make a material difference to the estimate of disease burden. A secondary aim is to employ assumptions derived from previous studies and empirical sources to assess the sensitivity of the YLD estimates of trichiasis with low vision and trichiasis with normal vision to uncertainty in the natural history model in terms of both the duration of different states of trichiasis and their disability weights.

## Methods

### National prevalence estimates

The detailed methods for the population based trachoma prevalence surveys have been described elsewhere [7]. In brief, surveys were conducted in ten districts in South Sudan between September 2001 and May 2005. Using a two-stage cluster random sample survey design a total of 23, 139 people were examined for trachoma signs using the WHO simplified grading system [8].

Visual acuity testing was conducted in Makiem payam district to assess distribution of vision status in people presenting with trichiasis. Three stages of vision loss were recorded according to the level of presenting visual acuity (Table 1). A two-stage cluster sampling method was used to select the households for visual acuity (VA) testing. Villages were selected in the first stage and households in the second. A total of 3,567 present members of the selected household were tested for VA. VA testing was conducted using the Snellen E chart at 6 meters. Those with VA<6/60, were evaluated with the Snellen chart at 3 meters and those with VA<3/60 were evaluated by counting fingers, hand movement and light perception as appropriate. An ordinal logistic regression model to the observed VA data was used to explore the age and sex distribution of the three categories of vision status in participants with trichiasis: normal vision; low vision; and blindness. All participants in whom trichiasis and cataract was identified were excluded from analysis; however, it was not possible to adjust for visual impairment due to other causes such as refractive errors [9], [10].

**Table 1:** Definition of vision status adopted from the international statistical classification of diseases (Source: WHO [30]).

Condition	Definition
Blindness	Visual acuity of <3/60 in the better eye
Low Vision	Visual acuity of <6/18 but ≥3/60 in the better eye
Normal Vision	Visual acuity of ≥6/18 in the better eye

Age-grouped and sex specific prevalence of trichiasis was estimated from the cross-sectional surveys and a logistic curve fitted to smooth the prevalence estimates across age groups. The prevalence of vision status in the sample population was calculated by multiplying the predicted probability of each vision status with the overall prevalence of trichiasis (9).

### Demographic data

Abridged life tables for Sudan for the year 2001 were obtained from the WHO Statistical Information System (WHOSIS) for males and females separately [11]. Demographic estimates for South Sudan are based on model life tables because vital registration data are either poor or not available. Life tables were collapsed to represent 5-year age groups from age zero to 75 years and above. Years Lived with Disability (YLD) estimates were estimated using prevalence proportions in South Sudan applied to one third of the total Sudanese population (South Sudan made up about one third of the total population of Sudan in 2001).

### Relative risk of mortality

Like the authors of the Global Burden of Disease study in 2000, we maintained the same assumptions here and applied relative risks of 2.5 and 1.5 of death for blindness and low vision respectively [12].

### Remission

The disabling sequelae of an established trachoma infection do not remit unless treated early enough. It was assumed, therefore, that the population has no access to treatment and that there is no remission once trichiasis has developed.

### Modelling the incidence and duration of trichiasis

The public-domain disease modelling software, DisMod II [13], was used to model trachoma in South Sudan and estimate unavailable parameters. DisMod II is a generic mathematical disease model which describes the relation between incidence, prevalence and mortality (IPM model) and can be used to supplement observational data producing internally consistent epidemiological estimates. Here the prevalence of each of the three stages of trichiasis in the district of Mankien, as well as assumptions about the remission rates and relative risks of mortality, were used to generate the estimated incidence and duration of each stage of the condition for the population of South Sudan.

Incidence was first calculated for blindness using the prevalence, the assumed relative risk of mortality of 2.5 and a remission rate of zero. While the only exit from blindness from trachoma is death, low vision has two exits, death and blindness. DisMod models only a single disease specific exit (case fatality) from the prevalent state, therefore to account for these double exits the incidence of blindness was added to the case fatality for trichiasis with low vision. Similarly, the same logic was applied to trichiasis with normal vision which we assumed had no excess mortality, by setting the case fatality of trichiasis with normal vision in the DisMod model equal to the incidence of trichiasis with low vision.

### Estimating Years Lived with Disability

Years Lived with Disability (YLD) estimates were calculated with the standard formula [14], [15]. Estimates were calculated with and without age weighting and discounting at 3%. Estimates presented here follow conventional age-weighting and discounting (3,1). Duration estimates for each age group and each disease stage were obtained from the DisMod II output.

### Duration

In our analysis we have used durations gained from two sources. For the first aim of our study, to assess the additional burden of trichiasis prior to loss of vision, we employed the durations obtained from modelling the observed prevalence, relative mortalities (or case fatalities) and remission rates using DisModII. For the second aim, the assessment of different assumptions about duration and disability weights, we have used two scenarios for both trichiasis with normal vision and with low vision using evidence gathered from literature.

Ghambir et al [16] reviewed all studies which reported an estimate of trachoma disease incidence and trachoma disease duration. Results varied greatly. In Tanzanian women, 27% of those with trichiasis had developed corneal opacity (CO) within 10 years [17]. In The Gambia 15% of a sample with trichiasis developed visual impairment or blindness in 12 years [18]. The range of durations cited in the literature suggests that environmental and contextual factors play an important role in the natural progression of the disease [16].

As the largest study of its kind, the estimates of transition observed by Munõz et al [17] were used as a basis here to make some assumptions about duration of trichiasis with normal vision. In their study of 4,898 women in Tanzania, 10-year cumulative incidence of CO from trichiasis was up to 35.1% in age groups under 35 and between 42.7% and 53.5% in age groups 35 and above. CO has not been further graded in the literature and there is no evidence to directly suggest duration of the low vision state. One study in The Gambia [18] found that over a 12 month period between 5% and 17% of those with incident CO transitioned to progressive CO.

From the above evidence we conservatively conjectured, that it could take about ten years for 50% of those 35 and above to transition to CO and about 20 years for those under 34 and younger to do so. We constructed an arbitrary range of durations positing that it may take between 15 to 25 years for 50% of those under 35, and between 5 and 15 years for those 35 and above, to progress from trichiasis with normal vision to trichiasis with low vision. It was also assumed that transition from low vision to blindness could take on average between 3 to 10 years (Table 2).

**Table 2:** Models used to assess the sensitivity of the burden estimates to uncertainty in the duration.

	Name	Mean Durations
Trichiasis with NV	Model 1a	15 years for ages <35 and 5 years for ages >35
	Model 2a	25 years for ages <35 and 15 years for ages >35
Trichiasis with LV	Model 1b	3 years for all age groups
	Model 2b	10 years for all age groups

NV – Normal vision.

LV – Low vision.

### Disability weights

The burden of trichiasis with normal vision has never been assessed before so it was necessary to derive a disability weight for the condition. Using DWs developed for other conditions for the GBD we have assumed the DW of trichiasis with normal vision to be within the range of 0.024 and 0.12. The lower and upper limit of this range are intended to represent consistent low level pain or discomfort and chronic severe pain respectively, while the pain and discomfort associated with Onchoceriasis is place as about equivalent to our mid-way DW of 0.068. .The DW used for trichiasis with low vision has varied between different burden studies. In order to reflect the uncertainty of the contribution of trichiasis with normal vision to total trachoma burden a range of DWs for trichiasis with low vision was also used corresponding to those used in previous studies (Table 3).

**Table 3:** Disability weights used to assess the sensitivity of the analysis to uncertainty in severity.

Source	Baseline (lower and upper limits)
Blindness	0.6
Low vision	0.245 (0.170–0.282)
Normal vision	0.068 (0.024–0.120)

## Results

### Estimating the Years Lived with Disability (YLD)

**Figure 1:**
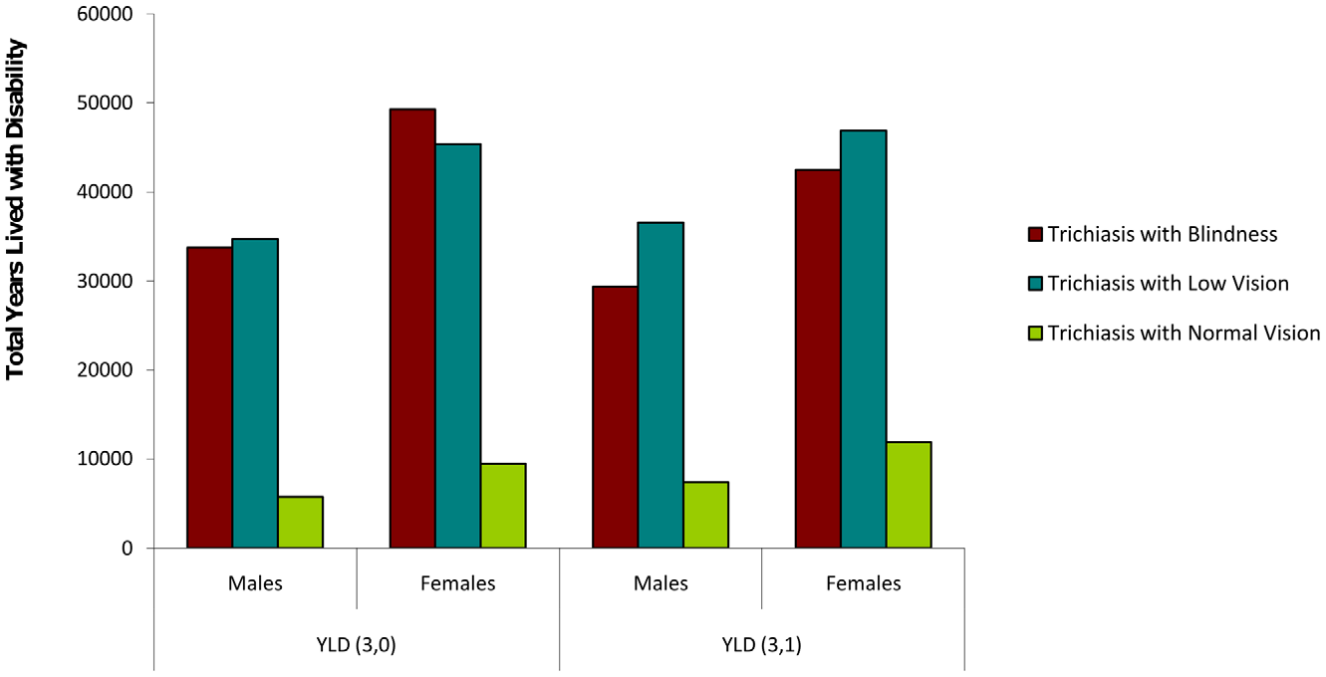
YLDs lost due to trichiasis with different levels of visual impairment in South Sudan. Assessed with (3,1) and without (3,0) age weighting (disability weights of 0.6, 0.245 and 0.068 for blindness, low vision and normal vision respectively).

Incidence rates and duration estimates for each of the trachoma disease states by age were modelled using DISMOD II and are presented here in Table 4. Employing these modelled durations and the disability weights of 0.245 and 0.068 for trichiasis with low vision and trichiasis with normal vision, respectively, all states of trachoma combined, incident in 1 year, resulted in 174,550 Years Lived with Disability in South Sudan (or 16.46 YLD/1000 person years). Trichiasis with normal vision contributes 19,219 YLDs, or 11%, to the total burden (Figure 1).

**Table 4:** Modeled incidence rates (/100) and durations by age for each disease state.

	Trichiasis with Blindness				Trichiasis with Low Vision				Trichiasis with Normal Vision			
	Males		Females		Males		Females		Males		Females	
Age	Incidence	Duration	Incidence	Duration	Incidence	Duration	Incidence	Duration	Incidence	Duration	Incidence	Duration
**0–4’**	0.00	42.10	0.00	44.91	0.01	49.56	0.01	52.76	0.02	53.35	0.04	55.83
**‘5–9’**	0.00	41.06	0.00	45.21	0.01	47.74	0.01	50.95	0.07	51.31	0.10	53.77
**‘10–14’**	0.00	37.31	0.00	41.02	0.02	43.19	0.03	46.49	0.09	46.97	0.14	49.45
**‘15–19’**	0.00	32.66	0.01	36.92	0.05	38.78	0.06	42.11	0.09	42.27	0.14	44.78
**‘20–24’**	0.01	29.12	0.01	33.13	0.08	34.73	0.09	38.05	0.10	38.11	0.17	40.61
**‘25–29’**	0.02	25.83	0.03	29.86	0.13	31.04	0.16	34.26	0.10	34.27	0.18	36.67
**‘30–34’**	0.04	22.96	0.05	26.79	0.23	27.63	0.28	30.68	0.12	30.65	0.21	32.97
**‘35–39’**	0.07	20.25	0.10	23.82	0.33	24.39	0.40	27.21	0.10	27.34	0.19	29.55
**‘40–44’**	0.13	17.47	0.17	20.78	0.46	21.10	0.54	23.67	0.08	23.94	0.16	26.06
**‘45–49’**	0.22	14.79	0.29	17.70	0.59	17.90	0.67	20.17	0.07	20.72	0.13	22.71
**‘50–54’**	0.36	12.26	0.45	14.77	0.72	14.85	0.77	16.82	0.05	17.66	0.09	19.49
**‘55–59’**	0.61	9.97	0.74	12.06	0.90	12.02	0.90	13.67	0.04	14.85	0.05	16.50
**‘60–64’**	1.10	8.00	1.26	9.61	1.22	9.52	1.11	10.79	0.02	12.36	0.02	13.91
**‘65–69’**	1.84	6.35	2.04	7.49	1.53	7.32	1.26	8.23	0.01	10.11	0.00	11.19
**‘70–74’**	2.95	5.16	3.18	5.93	2.20	5.68	1.69	6.33	0.01	8.15	0.00	8.44
**‘75+’**	5.51	2.66	6.70	2.52	3.21	3.56	2.46	3.52	0.07	4.84	0.09	4.20

Women carry more of the burden than males for all stages of the disease. The inclusion of trichiasis with normal vision contributes 11.73% to total burden experienced by females (with age-weighting) and is equivalent to 30% of the burden amongst women due to trichiasis with blindness.

**Figure 2:**
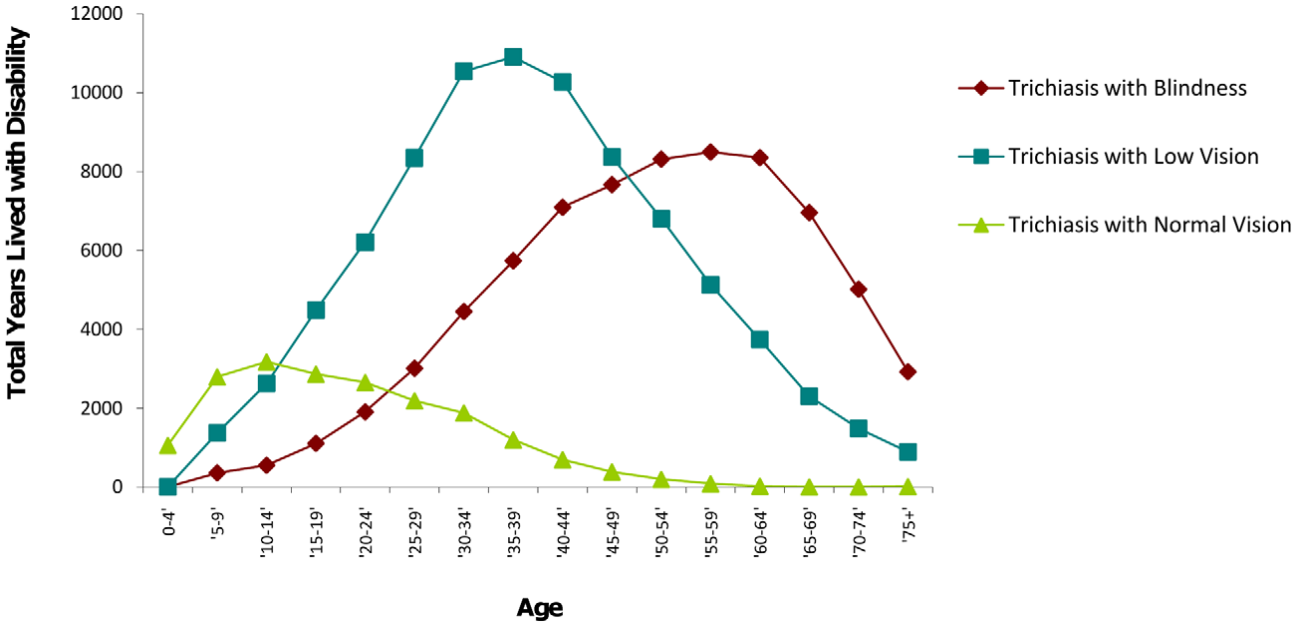
YLDs (3,1) lost due to trichiasis by age in South Sudan.

Most of the burden associated with trichiasis with normal vision is experienced during childhood and while the burden of trichiasis with low vision is placed upon young adults and blindness is mostly experienced in later life (Figure 2). Age weighting emphasizes the contribution of trichiasis with normal and low vision on the total burden of trachoma (Figure 1).

### Using alternative assumptions for uncertain estimates

Employing the different disability weights described above in Table 3, Table 5 reports the resulting range of Years Lived with Disability (YLD) estimates. Estimates of total YLD were sensitive to the application of different weights to trichiasis with both normal and low vision. The difference between the lowest and highest disability weight for trichiasis with normal vision was 5 times higher and 1.6 times higher for trichiasis with low visions.

**Table 5:** Years Lived with Disability/1000 person years (3,1) estimated using alternative disability weights.

	Total Years Lived with Disability(/1000 p-y)			
	Disability weights for trichiasis with normal vision			Difference between YLD estimates using the highest and lowest DW
	0.024	0.068	0.120	
Trichiasis with Normal Vision	**0.64**	**1.81**	**3.2**	**2**.**56**

	Disability weights for trichiasis with low vision			
	0.17	0.245	0.282	
Trichiasis with Low Vision	**5**.**46**	**7**.**87**	**9**.**06**	**3**.**6**

DW – Disability weight.

Depending on the disability weight employed, the burden due to trichiasis with normal vision can contribute between 5% and 21% of the total burden of trichiasis adding up to 33, 917 YLD to the total.

**Figure 3:**
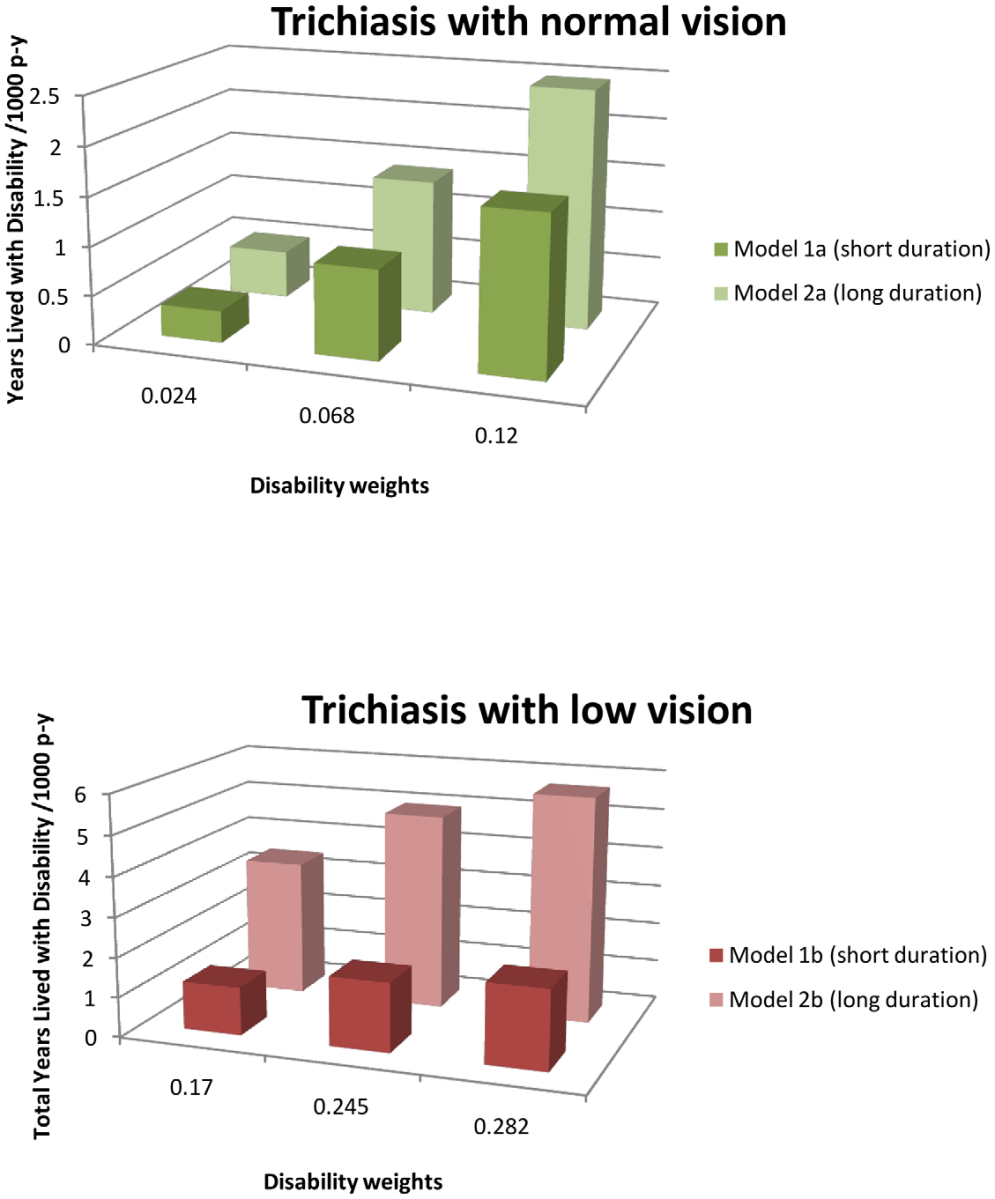
YLDs (3,1) (/1000 person years) using different assumptions about duration and severity in South Sudan.

Figure 3 presents the sensitivity of the results to the assumed durations of the two disease states. As would be expected, the longer the duration the more sensitive the results are to the different disability weights. Similarly, because trichiasis with low vision results in greater burden in South Sudan than trichiasis with normal vision, it exhibits more sensitivity to the assumptions employed.

Using the longer duration model 2a can add up to 0.84/1000 person years (or 8,883 YLD) for trichiasis with normal vision as compared with the short duration model 1a and using model 2b for trichiasis with low vision can add up to 3.69/1000 person years (or 39,171 YLD) as compared with the short duration model 1b (in both cases the highest DW have been applied).

## Discussion

Five attempts to calculate the global burden of trachoma have been conducted since 1990. The methodologies, parameter inputs and data sources used in each of these studies have not always been consistent and are not all directly comparable. The estimates suffer most from the paucity of reliable data on trachoma prevalence and incidence and the uncertainty surrounding the estimates used [19]. Furthermore, no data has previously described the prevalence of trichiasis pre-visual impairment.

In the present model trichiasis with low vision was the biggest contributor to the total burden of trachoma which reflects current knowledge. This work represents the first attempt to estimate the incidence and burden of trachoma without vision loss in a population and our analysis reveals that the inclusion of this state can potentially add a substantial amount to the total trachoma burden estimates.

Our results also indicated a decreasing incidence of this state with age in both males and females. Due to the small numbers in older age groups, however, we have refrained from drawing any conclusions on this observed trend

As noted elsewhere [10], females carry more of the burden of trachoma than males. This could partly be due to their longer life spans and thus a greater number of years spent with visual impairment. However, the woman's role in child caring and their relatively greater exposure to the reservoirs of bacteria carried by children does put them at greater risk of transmission and they experience higher rates of incidence for all states through most age groups. In fact the burden suffered by women, may more likely be underestimated in this study because we assumed that females had both an equal risk of mortality due to blindness and low vision as males as well as equal experiences of severity. Some evidence suggests, however, that females affected by visual impairment may experience a higher risk of death to men (3.8 and 1.5 respectively) [20] and also suffer from greater functional limitation prior to visual impairment [5].

### Duration and disability weights

Valuing health states is a contentious business and disability weights have attracted a fair amount of critical attention since the development of the DALY. One of the major challenges in developing disability weights arises when a disease is characterized by a spectrum of severity levels and when there are multiple stages of differing severity. A series of efforts have attempted to construct DWs capable of capturing this disability by foregoing the assumption underlying the standard DALY which requires independence between duration and disability [21]–[23]. The secondary aim of this study was to assess the impact of using both a range of disability weights and durations. Many of these assumptions were arbitrary and were not scientifically derived but they were intended to show the extent to which a range of inputs, providing a reasonable reflection of the uncertainty around these parameters, may impact upon the outcome of such an analysis. In the case of trachoma in South Sudan, the burden estimates are particularly sensitive to the range of durations employed here.

### Conclusions

Regardless of the assumptions used, the health burden of trachoma is consistently under-estimated because the disease state of trichiasis without vision loss had not previously been included in estimations. Including this state increases burden estimates considerably. Underestimating the disease burden caused by trachoma understates its importance not only to those directly affected but also to those at risk. Understating the burden leads to neglect of disease; not recognizing the impact of successful control measures and not prioritising trachoma control for neglected populations.

Burden estimates of a number of diseases classified as ‘neglected tropical diseases’ have been revisited recently. The burdens estimated for schistosomiasis, leishmaniasis, diarrhoea and rabies for example have all been re-assessed with the aim of ensuring that the complex patterns of disability characteristic of these conditions is taken into account [24]–[29]. In this study we have looked at a disease that is characterised by graded severity and noted that the priority is for more robust estimates of disease duration. Until a better understanding of trachoma's natural history is gained, efforts to perfect the disability weights to be assigned to the different severity levels will be mostly wasted [23]. Estimating disease burdens is an iterative process which requires on-going, self-assessment and self-correction as more data become available.

In the case of trachoma, as with other non-fatal conditions, there remains considerable uncertainty around the occurrence parameters, and the duration of different stages of the disease in particular. Improving these estimates remains a priority.
